# The San Antonio Meeting

**Published:** 1899-05

**Authors:** 


### Editorial Department.

F. E. DANIEL, M. D., Editor.
S. E. HUDSON, M. D., Managing Editor.
A. J. SMITH. M. D., Galveston, Associate Editor.

Published Monthly at Austin, Texas, by Drs. Daniel and Hudson. Subscription price $1.00 a year in advance.

Eastern Representative: John Guy Monihan, St. Paul Building, 220 Broadway, New York City.

Official organ of the West Texas Medical Association, the Houston District Medical Association, the Austin District Medical Society, the Brazos Valley Medical Association, the Galveston County Medical Society, and several others.

### The San Antonio Meeting.

The Thirty-first Annual Meeting of the Texas State Medical Association, held at San Antonio, April 25, 26, 27 and 28 (ult.), was a most gratifying success, from every point of view,—scientifically, socially and numerically. There were about two hundred and fifty physicians in attendance, and many of them were accompanied by lady members of their families. The latter, be it understood, were not in attendance upon the *Convention,* but came in response to the whole-hearted invitation of the hospitable people of the grand old historic city to partake of its hospitality, to see the world famed "Alamo" and the "Missions," and to enjoy the delights of spring in that garden of roses. Interesting at all times, San Antonio is simply charming in the first flush of spring. A splendid program had been arranged, and was carried out as fully as time would permit. There were a great many papers read and discussed, and some were of high scientific value. The program was so full that many papers were simply read by title, for want of time, and referred to the Publishing Committee. They will appear in the annual volume of Transactions in due time. The Journal will also publish some of them.

The meeting will be long remembered by those who were fortunate enough to be present. The Association has awakened to a realization that if anything is ever to be accomplished in the way of sanitary or medical legislation the profession must perfect its organization. The Journal has insisted upon this for many years, and we are gratified to see that steps were recommended by the president and adopted looking to organizing the profession in each county, precisely in accordance with the suggestions repeatedly made by the JOURNAL. The following members were appointed a Committee on Organizing County Societies:

Dr. S. C. Red, Houston.
Dr. J. C. Erwin, McKinney.
Dr. J. A. Rawlings, El Paso.
Dr. J. R. Nichols, Terrell.
Dr. W. Shropshire, Yoakum.

Mayor Marshall Hicks welcomed the Association in a pretty speech: Judge Ogden did the same: Dr. J. H. Bell, of San Antonio, extended welcome in the name of the West Texas District Medical Association. To all of which President Wilson responded in appropriate terms.

Then began the routine work, calling the roll, reports of Secretary and Treasurer, reports of committees, etc.

ANNUAL MESSAGE AND RECOMMENDATIONS.

President Wilson then delivered his annual message and recommendations, as follows:

"GENTLEMEN: It is with much pleasure that I greet you on this auspicious morning of the beginning of the Thirty-first Annual Session of the Texas State Medical Association, and wish with all my heart that you may have a most pleasant and enjoyable visit to this interesting city, and a satisfactory and profitable meeting.

"Under the previous administration the financial condition of the Association was so much improved that no debt was allowed to hang like a pall—as all debts do—over its doors, and when the year closed a small balance was left in the treasury. It seems as if the harmonious and successful meeting one year ago at Houston was propitious of a new era of the Association, and the future has bright promises in store for it. During the past year the Secretary has produced a beautiful volume of the Transactions, and in addition, it being an unusually expensive year, yet, the Treasurer has been able to pay all dues and there still remains a balance of over $400 in the treasury. I would respectfully recommend that the same business methods of collecting dues and dispensing the funds which has proven so successful and satisfactory in the last two years be continued, and a cash basis be maintained.

"In view of the increase of nervous diseases and insanity in our

State, and the necessity for a more extended study by the profession of these diseases, I beg to recommend for your attention the advisability of adding to the by-laws a section on 'Neurology and Mental Diseases.'

"Medical legislation is still an important subject for consideration by this body. Neither the work of the Committee on the Medical Examining Board or that of the Board of Health, has met or is likely to meet with any degree of success during the session of the present Legislature. This is owing to a want of a better organization of the profession of the State, to the want of zeal by the majority of its members, and the lack of unity upon the measures suggested—reports of the committees on the subjects will enter more into details. I would most earnestly suggest that this body do not cease in its labors in this direction, but on the other hand to continue the work with unabated zeal. In view of the opposition to the medical legislation, I am inclined to the belief that it will be the part of wisdom to determine upon one subject at a time, concentrate the united strength of the profession upon it, and labor for its success; when that has been accomplished, take up the other with the same enthusiastic determination to have it enacted into a law. Taking into consideration that opposition to the creation of a Board of Health is greater than that of a Medical Examining Board, and besides the latter would necessitate no demand on the treasury, there being strong opposition to new laws which require an appropriation for their maintenance, it might be wise to first unite upon a bill for the creation of a Medical Examining Board, which would cost the State nothing.

"I beg to reiterate the recommendations of my predecessor in regard to the organization of county societies all over the State. I would recommend that a committee of five or seven, as may seem expedient, be appointed to attempt this organization. If a good working committee be appointed, they can, by correspondence, interest the medical men of the various counties, induce them to affiliate with their nearest society, and when practicable, form new organizations. Urge each society to send delegates to and become identified with this body. This will effect a more complete organization of the State, serve in a measure to unify the profession, and constitute a power for good. Without organization and harmony, nothing can be accomplished.

"Let us continue our efforts in making this Association a scientific working body—spending its time in section work, where young and old alike can come and drink from its fountain of wisdom and experience. Let those devoted men who spend their time in preparing good papers feel that they have not labored for naught; that they can confidently come here with the assurance of an opportunity to read them, and listen to the discussions. Let the time be so systematized that every paper brought here may be read, and an opportunity given for discussion on it. Let it be the ambition of every member to lend his aid in contributing to the usefulness of this Association by promoting its scientific work, and then busy men will be attracted to it. It will grow rapidly in favor, in that way assist the organization of local societies and invite their affiliation, and thus its influence will be felt in State medicine and all other subjects pertaining to the art.

"I would respectfully call your attention to some resolutions passed by the American Medical Association at its meeting in Denver, in June last, affecting the standing of medical men in regard to their relations to that body. I would suggest that these resolutions, as read, receive some consideration of the committee who will take charge of them, and take such action as in its wisdom may seem appropriate.

"And now comes the saddest part of my duty. The year just passed has stricken from the rolls of this Association some of its most honored names. Among the honorary members whom death has claimed, we find Dr. David W. Yandell, of Louisville, Ky., conspicuous in the history of surgery in the west, who passed away on May 3, 1898; Dr. J. J. Noyes, Providence R. I.; Dr. Thos. G. Heard, Galveston, Texas, March 8, 1899; and among the active members who will answer no more are Dr. D. Dupree, of Dallas, November 28, 1898, and Dr. R. M. Swearingen, Austin, Texas, August 7, 1898. While I mourn the loss of all these distinguished men, I cannot pronounce the name of the latter without a sigh. Dr. Swearingen had been my warm personal friend for twenty years, and I loved him as a brother. The ripe scholar, the learned physician, the brilliant orator, the warm-hearted gentleman, he was in all respects a man. Ever at the post of duty, he was ever ready to respond to the call of suffering humanity, and his generous heart never failed in its sympathy for the distress of others. The hut with its squalid poverty and the palace with its rich luxury received alike his valued services. Once the honored President of this Association, he was worthy of and wore with modesty all the honors conferred upon him. I pause to lay my humble tribute upon his grave, to weave a wreath of immortelles to his memory. Peace to his ashes.

"I beg to thank you, gentlemen, for the kind consideration and courtesy to me, to assure you of my high appreciation of your confidence and partiality."

REPORT OF THE SECRETARY.

Dr. H. A. West, the Secretary, read his annual report, which is as follows:

*"Mr. President and Gentlemen.*

"I beg to present herewith my annual report, and incorporate with the same a statement concerning publication of the Transactions for 1898:

*"Membership on the Roll of Transactions of 1898.*—Honorary, 32; ordinary, 323; total, 355. Elected at Houston, April 26-29, 1898—Honorary, 6; ordinary, 64; total, 70. Dropped from the roll since 1897—By death, 3; by non-payment of dues, 3; transferred from ordinary to honorary roll, 3; reinstated, 19.

*"Deaths Since Last Report.*—Dr. R. M. Swearingen, of Austin, died August 7, 1898; Dr. D. Dupree, of Dallas, died November 27, 1898; Dr. J. F. Noyes, of Providence, R. I., date of death not reported; Dr. David Yandell, of Louisville, Ky., died May 3, 1898; Dr. Thomas G. Heard, of Galveston, March 8, 1899. There may have been other deaths to which my attention has not been called.

*"The Transactions for 1898.*—Funds were in the Treasurer's hands ample to meet the cost of publication, hence the work was commenced as soon after the Houston convention as possible, and completed about the middle of October. The lowest bid received was that of the Von Boeckmann Publishing Company, of Austin, which was accepted.

"It affords me much pleasure to report that the adoption of strict business methods as regards collection of dues, and the restoration of a small initiation fee, has resulted in an immense improvement of the financial status of the Association. We are not only out of debt, but the Treasurer's books show a larger balance than at any previous time within my knowledge. While the membership is not as large as it should be, there is certainly no ground for the groanings of the perennial pessimists, who so unceasingly bewail the 'decline and fall' of the Association that one is tempted to believe the 'wish is father to the thought.'

"The immense area of Texas and the large number of physicians within her borders, while apparently a source of strength, is in reality a cause of numerical weakness in the State Association, for this condition has encouraged the organization of various large district societies—the North, South, East, West and Central, all of which flourish to a certain extent at the expense of this body, for many physicians are satisfied with the advantages they obtain from connection with their local societies, which are secured at a minimum cost both of time and money. Our growth, development and usefulness, in my humble judgment, depends upon a better esprit de corps, which will grow out of a more vital connection with the various local societies.

"We have recently had another object lesson illustrating our want of influence in the failure to secure adequate legislation in matters relating to the public health. Without attempting to discuss in extenso the reasons for our want of success along these lines, let me express my earnest conviction that when the united power and intelligence of the State Association, with all of its affiliating societies, is brought to bear upon the executive and law makers, that we will accomplish the grand object of our organization, but so long as the relations between those bodies is nominal only, we will fail as in the past. As the suggestions I have heretofore made to remedy this condition have not been acted upon by the Association, I will not at this time urge further consideration of the subject."

REPORT OF COMMITTEE ON MEDICAL LEGISLATION.

The Committee on Medical Legislation, to whom the duty of submitting to the Legislature a bill providing for State Boards of Medical Examiners for Texas was entrusted, composed of Drs. A. B. Gardner, Z. T. Bundy, L. Ashton, Taylor Hudson, and J. T. Wilson, reported that the bill was put in charge of Dr. Yett, of the Senate, who worked so faithfully for the bill adopted by the Association three years ago, and he having been appointed chairman of the Committee on Public Health, to whom all such matters are referred, it was believed that he was interested in the public welfare, was patriotic in his motives, and would be the most appropriate person in that body to have charge of it. In consulting with his committee and finding some opposition to the bill, he declined to introduce it. Information of this fact came too late to seek another channel for its introduction in the Senate.

"The bill was introduced in the House by Hon. Cecil Smith, the able, conservative member from Grayson county. It was referred, as is the usual course, to the Committee on Public Health, on which were three physicians. When that bill, with two others which had been introduced, were taken up for consideration, your committee, in company with a number of able and prominent members of this

Association, were granting the privilege of appearing before that committee, making a statement in explanation of the subject, and replying in a succinct way to all questions asked. It is due to state that this committee, and members accompanying it, were accorded all due courtesy by the Public Health Committee.

"It was learned that after the members of this Association retired the two bills first read were killed, and a subcommittee appointed to take into consideration the Association bill and report upon it in a week. It was afterwards learned that the Association bill was dropped, and an entirely new bill reported and acted upon favorably. It was also learned that instead of its being introduced in the House, it was entrusted to Senator Davidson, introduced by him in the Senate, and was called the Davidson bill. Taking the usual course, it was reported back favorably by the Public Health Committee of the Senate to that body, and rests there, and this committee hopes it will continue to so rest.

"It is but due to state that the members of this committee worked faithfully in the discharge of their duty, interviewed members of this Association, and that the profession rendered valuable services in the fight.

"When the bill was fully explained, the legislators would almost invariably express themselves as being favorable to it, confessed that such a law was badly needed, and we believe that if the Committee on Public Health had reported it back favorably it would have passed both houses. Nearly all members approached would state that 'the doctors in the Legislature are opposed to it, and that fact will make it difficult to pass it.'

"It was soon learned that there was a strong undercurrent of opposition among the doctors in the Legislature, and a few ex-members of this Association, and that, gentlemen, is undoubtedly the cause of the defeat. It is a notable fact that not one of the doctors in the Legislature is a member of this Association, and reading between the lines it can be readily seen that they are opposed to any kind of medical legislation and not interested in the progress of medicine, nor manifest that pride which every lover of science should feel in the welfare of his profession.

"This committee begs leave to recommend that this Association continue with untiring enthusiasm in this work, believing it to be the duty of this body to persist in laboring for the good of society by insisting upon the better average equipment of medical men, protect the people from ignorance, quackery and charlatanism; that it keep in line of progress with other States and other countries in the progress of the art and science in State Medicine.

"We beg to recommend that a committee of five be appointed to draft with care another bill for the creation of a Board of Medical Examiners for Texas, and submit to this body at its next annual meeting."

President Wilson, when he had finished reading the report, read a part of "A bill to be entitled an act to regulate the practice of medicine and surgery; to license physicians, surgeons and midwives, and to punish persons for violating the provisions thereof in the State of Texas."

The committee was continued, the report adopted, and the (substitute) bill before the Legislature was condemned by a unanimous vote of the convention.

Secretary Dr. H. A. West, of Galveston, offered a resolution in regard to the medical bill known as the Davidson bill, now pending before the State Legislature, which was unanimously adopted, as follows:

"That the report of the Committee on Legislation be received, and, since that committee has been paying special attention to this work, that said committee be continued; that this convention endorse the action of the committee and its chairman, Dr. Wilson, and condemn this hybrid monstrosity in the shape of a medical bill in toto, and express it as our opinion and sentiment that we had better have no bill, or the present bill, than such a monstrosity as the bill known as the Davidson bill."

Dr. McGregor presented a resolution that the Association request such legislation as will protect the people from the evils of morphine, cocaine, and kindred narcotics, by confining its distribution to orders from physicians, and that all proprietary medicines or drugs have printed upon the label in plain letters the formula of contents of the same.

This brought out a discussion, in part as follows:

Dr. Gardner said: "I am opposed to any resolution, memorials or lobbying in any way with the Legislature of Texas for anything relating to the practice of medicine, or anything emanating from this body going before the Legislature. Not that I feel that we do not need medical legislation, and this would be a good bill, but I am moved to oppose this action out of respect for this Association.

We have never accomplished anything, and have hardly been accorded a respectful hearing. We have been spurned. We can accomplish our end only by advance pledges from legislative candidates."

Dr. Denton said: "I endorse what Dr. Gardner has said. I do not see the necessity of appointing a committee when the Legislature is on the eve of adjournment. It will receive no attention. I am opposed to beseeching on our knees the Legislature of Texas for legislation that the people are interested in more than the medical profession."

Dr. Sears: "There is in Scripture 'an unjust judge, who had no respect for God nor man,' like our State Legislature, but often importuned finally did justice. I think we ought to ding-dong at the State Legislature a hundred years."

Dr. Lemond said that Denver had been headquarters for "quackery," but that after persistent efforts on the part of the fraternity, had passed a bill resulting in the complete expulsion of quackery.

Dr. Walker, Dr. Paine, Dr. Bell, Dr. Ghent, and Dr. Red, also took part in the discussion.

Dr. McGregor's resolution was adopted.

Forty-five new members were added to the roll. The Transactions are paid for, the Association is out of debt, and has money on hand.

Dr. J. Larendon, who has been Treasurer more than twenty-five years, insisted on resigning. His resignation was reluctantly accepted, and a complimentary resolution of thanks to him was adopted by a rising vote.

The following is a complete list of the officers elected and committees appointed:

President, Dr. A. B. Gardner, Bellville; First Vive-President, Dr. B. E. Hadra, San Antonio; Second Vice-President, Dr. George H. Lee, Galveston; Third Vice-President, Dr. F. D. Thompson, Fort Worth; Secretary, Dr. H. A. West, Galveston (holds over); Treasurer, Dr. R. F. Miller, Sherman.

Judicial Council.—Dr. J. J. Robert, Hillsboro; Dr. W. R. Blailock, McGregor; Dr. C. M. Alexander, Coleman; Dr. S. E. Hudson, Austin.

Orator.—Dr. Joe D. Becton, Greenville.

Publishing Committee.—Dr. H. A. West, Secretary, Chairman,

Galveston; Dr. J. F. Y. Paine, Galveston; Dr. H. P. Cooke, Galveston.

Committee on Necrology.—D. J. C. Erwin, Coleman; Dr. P. C. Coleman, Colorado; Dr. R. H. Harrison, Columbus.

The following delegates were elected to the American Medical Association Convention, which will be held at Columbus, Ohio, this year: Dr. J. O. Norris, Eagle Lake; Dr. M. M. Smith, Austin; Dr. J. R. Stuart, Houston; Dr. W. H. Monday, Terrell; Dr. R. B. Grammar, Fort Worth; Dr. H. A. West, Galveston; Dr. W. W. MacGregor, San Antonio; Dr. R. F. Miller, Sherman.

Waco was chosen for next meeting, fourth Tuesday in April, 1900, and Dr. J. H. Sears, of Waco, was made chairman of the Committee of Arrangements.

The committee consisting of the President and Vice-Presidents made the following appointments for the sections for the next meeting:

General Medicine.—Dr. H. P. Cooke, Galveston, chairman; Dr. S. C. Red, Houston, secretary.

Obstetrics and Diseases of Children.—Dr. J. J. Robert, Hillsboro, chairman; Dr. S. R. Burroughs, Buffalo, secretary.

Surgery.—Dr. B. F. Kingsley, San Antonio, chairman; Dr. J. R. Stuart, Houston, secretary.

Medical Jurisprudence.—Dr. I. E. Clark, Schulenburg, chairman; Dr. A. N. Denton, Austin, secretary.

State Medicine and Public Hygiene.—Dr. J. W. Shearer, Wallisville, chairman; Dr. J. W. Scott, Houston, secretary.

Gynecology.—Dr. T. J. Bell, Tyler, chairman; Dr. C. M. Rosser, Dallas, secretary.

Ophthalmology, etc.—Dr. W. F. Cole, Waco, chairman; Dr. J. O. McReynolds, Dallas, secretary.

Pathology.—Dr. W. R. Blailock, McGregor, chairman; Dr. J. M. Frazier, Belton, secretary.

To present subjects: General Medicine.—Dr. J. D. Osborne, Cleburne; Dr. J. C. Erwin, McKinney, to open discussion.

Surgery.—Dr. Bacon Saunders, Fort Worth; Dr. I. P. Gunby, Sherman, to open discussion.

Obstetrics and Diseases of Children.—Dr. Taylor Hudson, Belton; Dr. C. M. Alexander, Cleburne, to open discussion.

State Medicine and Public Hygiene.—Dr. M. M. Smith, Austin; Dr. F. E. Daniel, Austin, to open discussion.

Gynecology.—Dr. S. E. Hudson, Austin; Dr. R. R. Walker, Paris, to open discussion.

Ophthalmology.—Dr. R. E. Moss, San Antonio; Dr. H. L. Hilgartner, Austin, to open discussion.

An excursion over the Aransas Pass Railroad to Rockport, thence by sail boat to Aransas Pass, was given, in honor of the delegates and their ladies, and was enjoyed by some two hundred of the city's guests. At Aransas Pass they were the guests of the "Tarpon Club," a swell organization, and were entertained at a wine and fish dinner—a most sumptuous affair—with toasts and responses galore. Ye tired editor didn't go, so he can't tell you anything about it except that many returned with red noses, but they said it was sunburn.

The occasion—long to be remembered—closed with the swellest kind of a "reception," a ball and supper, at which there were not less than five hundred persons. It was given by the leading society ladies of San Antonio, and was a most brilliant and delightful "function."

President Wilson's Address was a scholarly effort, the subject being the History and Progress of Medicine.

D. Lemond, of Denver, Colorado, was the only visitor from another State.

